# Loss of *stra8* Increases Germ Cell Apoptosis but Is Still Compatible With Sperm Production in Atlantic Salmon (*Salmo salar*)

**DOI:** 10.3389/fcell.2021.657192

**Published:** 2021-04-16

**Authors:** Kai O. Skaftnesmo, Diego Crespo, Lene Kleppe, Eva Andersson, Rolf B. Edvardsen, Birgitta Norberg, Per Gunnar Fjelldal, Tom J. Hansen, Rüdiger W. Schulz, Anna Wargelius

**Affiliations:** ^1^Institute of Marine Research, Research Group Reproduction and Developmental Biology, Bergen, Norway; ^2^Institute of Marine Research, Research Group Reproduction and Developmental Biology, Austevoll Research Station, Storebø, Norway; ^3^Institute of Marine Research, Research Group Reproduction and Developmental Biology, Matre Research Station, Matredal, Norway; ^4^Reproductive Biology Group, Division Developmental Biology, Department Biology, Science Faculty, Utrecht University, Utrecht, Netherlands

**Keywords:** stimulated by retinoic acid 8, gene editing, spermatogenesis, apoptosis, single cell proliferation

## Abstract

Entering meiosis strictly depends on *stimulated by retinoic acid 8* (*Stra8*) gene function in mammals. This gene is missing in a number of fish species, including medaka and zebrafish, but is present in the majority of fishes, including Atlantic salmon. Here, we have examined the effects of removing *stra8* on male fertility in Atlantic salmon. As in mammals, *stra8* expression was restricted to germ cells in the testis, transcript levels increased during the start of puberty, and decreased when blocking the production of retinoic acid. We targeted the salmon *stra8* gene with two gRNAs one of these were highly effective and produced numerous mutations in *stra8*, which led to a loss of wild-type (WT) *stra8* expression in F0 salmon testis. In maturing *stra8* crispants, the spermatogenetic tubuli were partially disorganized and displayed a sevenfold increase in germ cell apoptosis, in particular among type B spermatogonia and spermatocytes. The production of spermatogenic cysts, on the other hand, increased in maturing *stra8* crispants. Gene expression analysis revealed unchanged (*lin28a, ret*) or reduced levels (*egr1, dusp4*) of transcripts associated with undifferentiated spermatogonia. Decreased expression was recorded for some genes expressed in differentiating spermatogonia including *dmrt1* and *ccnd2* or in spermatocytes, such as *ccna1*. Different from *Stra8*-deficient mammals, a large number of germ cells completed spermatogenesis, sperm was produced and fertilization rates were similar in WT and crispant males. While loss of *stra8* increased germ cell apoptosis during salmon spermatogenesis, crispants compensated this cell loss by an elevated production of spermatogenic cysts, and were able to produce functional sperm. It appears that also in a fish species with a *stra8* gene in the genome, the critical relevance this gene has attained for mammalian spermatogenesis is not yet given, although detrimental effects of the loss of *stra8* were clearly visible during maturation.

## Introduction

In mammals the start of meiosis strictly depends on *Stra8* (Endo et al., 2015), and loss of this gene in mice leads to sterility in both males and females (Baltus et al., 2006; Anderson et al., 2008). STRA8 activates a broad transcriptional program in the mouse testis, that includes genes involved in the meiotic prophase, but also DNA replication and the G1-S cell cycle transition (Kojima et al., 2019). Considering that *Stra8* transcripts were detectable already in undifferentiated spermatogonia in mice (Zhou et al., 2008), STRA8 may have functions, although perhaps not critical ones, also during the early mitotic phase of spermatogenesis. Retinoic acid (RA), a derivative of vitamin A, strongly up-regulates expression of *Stra8* in mammalian germ cells which is critical for developmental transitions in spermatogenesis including entry into meiosis (Endo et al., 2015). Also in teleost fish which lack a *stra8* gene (Pasquier et al., 2016), RA signaling promotes spermatogenesis such as the differentiation of spermatogonia in zebrafish (Crespo et al., 2019a), or the meiotic initiation in medaka (Adolfi et al., 2016) and tilapia (Feng et al., 2015). As these fish still are able to successfully complete spermatogenesis despite lacking a *stra8* gene, the functional relevance for *stra8* in fish needs further investigation. Zebrafish, medaka and tilapia belong to the two teleost fish taxa (*Acanthomorpha* and *Cypriniformes*) having lost the *stra8* gene, while *stra8* has been identified in at least fourteen other teleost fish including Southern catfish (*Ictalurus punctatus*) and three salmonids [*Oncorhynchus mykiss*, *Salmo trutta* and *Salvelinus alpinus* (Pasquier et al., 2016)], but there is little information on *stra8* functions in fish. In catfish, *stra8* is expressed in germ cells of both sexes, and functional experiments showed that RA, as in mammals, activated *stra8* expression and meiotic initiation in the ovary (Dong et al., 2013; Li et al., 2016). However, direct evidence regarding *stra8* functions in teleosts is currently missing. We therefore wanted to explore *stra8* functions in Atlantic salmon, a fish species which has retained a copy of the *stra8* gene. We here posed the following questions: is *stra8* required for meiosis/fertility in Atlantic salmon males, as in mammals? If *stra8* is not required, is this gene relevant at all in salmon spermatogenesis, and is a major RA function in mammals, the up-regulation of *Stra8* gene expression, detectable in fish?

To address these questions we examined testis of salmon *stra8* crispants histologically, quantified germ cell proliferation and apoptosis as well as analyzed a repertoire of genes involved in spermatogenesis. Testicular *stra8* expression was nearly completely lost in *stra8* crispants, correlating well with the high mutation rate found in F0 fish. Male crispants entered puberty but displayed disorganized spermatogenic tubules with an elevated incidence of apoptosis, in particular among type B spermatogonia and spermatocytes, during the rapid testis growth phase. This clear phenotype disappeared during the further progress through maturation. Potentially related to the increase in the production of spermatogenic cysts observed in crispants, we observed that in both, crispants and wild-type (WT) controls, testis weight did not differ, many germ cells completed spermatogenesis and sperm was able to fertilize eggs.

## Materials and Methods

### Guide RNA Design and Synthesis

High scoring guide RNA (gRNA) target sequences were predicted using CRISPRscan (Moreno-Mateos et al., 2015). Templates for producing gRNAs were then made according to protocols published by Gagnon et al. (2014) with minor modifications. Two partially complementary oligos, one forward gRNA oligo that encompasses the T7 polymerase II promoter, a 20 bp sequence that targets DNA and a 15 bp constant region that primes into a common reverse primer that contains the gRNA scaffold, were annealed and the protruding ends were filled in with a T4 polymerase to produce a full double stranded template. Templates were purified using the QIAGEN PCR purification kit and used as input for an *in vitro* transcription reaction using T7 HiScribe high yield kit (NEB). *In vitro* transcribed gRNAs were then treated with the TURBO DNA-free kit (Thermo Scientific) to remove template DNA, and purified using the QIAGEN RNeasy kit with a protocol modified for purification of small RNAs. Two gRNAs were prepared targeting the 5′-end and 3′-end of *stra8*, respectively (oligonucleotide sequences are shown in Figure 2A). Cas9 mRNA was prepared as previously described (Edvardsen et al., 2014).

### Microinjection

Microinjection of fertilized and glutathione-softened salmon eggs was performed as previously described (Edvardsen et al., 2014). Two gRNAs targeting the 5′- and 3′-ends of *stra8* were co-injected with a gRNA targeting *slc45a2*, aiding the selection of mutated individuals due to the loss of pigmentation (Wargelius et al., 2016). Sibling fish was kept as a control group.

### Animal Housing and Tissue Sampling

The fish used in this study were reared and sampled at Matre Aquaculture Research Station (Matredal, Norway). The eggs used in the experiment were fertilized in November 2015, and full or partial albinos (*slc45a2* knockouts) as well as sibling controls were started under standard conditions. In August 2016, a PIT-tag was implanted to allow individual identification of the fish, and a fin-clip was taken for genetic sex determination (Eisbrenner et al., 2014) and for analysis of mutation frequency with MiSeq. Equal numbers of sibling controls and *stra8* crispants were then mixed into a common garden.

To initiate early male maturation in postsmolt males, fish were exposed to a 6-week stimulatory period from January-March 2017 according to established protocols (Fjelldal et al., 2011). Four (May 9th) and 8 months (September 26th) after starting the maturation regime, samplings took place from mutant and WT groups. Gonad and blood samples were collected, and gonado-somatic indices (GSI) calculated from *stra8* crispants and WT fish. From the sampled fish, gonad tissue was collected in RNAlater (Thermo Fisher Scientific) for RNA extraction. In September 26th, three out of ten mature *stra8* crispants exhibited gonads containing white-colored areas – as observed for all WT and most crispant males – but also gray areas, reminiscent of the coloration of maturing testis tissue. Samples for the analysis of gene expression were only taken from the white-colored areas in order to investigate gene expression changes in comparable stages of spermatogenesis. Gonad tissue pieces for subsequent histological analyses, however, were collected from white- and gray-colored areas (in case of the three mature crispants). Tissue samples were fixed in 4% paraformaldehyde or 4% glutaraldehyde and embedded in paraffin or plastic (Technovit 7100, Kulzer), respectively, as previously described (Wargelius et al., 2016; Kleppe et al., 2017).

*In vivo* testicular expression of genes involved in spermatogonial differentiation (*sall4* and *dusp4*) and RA testicular function (*stra8* and *rec8*) was investigated in salmon males before and after starting pubertal development. For that purpose, testicular tissue was collected from immature and maturing postsmolt males (13–15 months old) exposed to maturation-inducing conditions (Fjelldal et al., 2011) for 16 days. In response to this regime, part of the fish had started pubertal development, as indicated by GSI levels above 0.1% and the presence of type B spermatogonia, respectively. Testis tissue was fixed in 4% glutaraldehyde overnight at 4°C and embedded in Technovit 7100 for histological evaluation or collected in RNAlater for RNA extraction. Moreover, *sall4*, *dusp4*, *stra8* and *rec8* mRNA levels were analyzed in testicular fragments collected from immature postsmolts and cultured in basal and RA-free conditions (see Testis tissue culture section).

This experiment was approved by the Norwegian Animal Research Authority (NARA, permit number 5741) and the use of these experimental animals was in accordance with the Norwegian Animal Welfare Act.

### MiSeq Analysis

Genomic DNA from fin clips were purified using the QIAGEN DNA extraction protocol. DNA was used in a two-step barcoding PCR targeting the two CRISPR locusts in *stra8* as described by Gagnon et al. (2014) An amplicon for each of the targeted regions was prepared. For the exon 3 region a forward primer: 5′-TCT TTC CCT ACA CGA CGC TCT TCC GAT CTG GGG CAG CAT CAA TTA GCT T-3′ and reverse primer: 5′-TGG AGT TCA GAC GTG TGC TCT TCC GAT CTT GTC ATT CTG AGC ACC GTG G-3′ was used. For the exon 6 region a forward primer: 5′-TCT TTC CCT ACA CGA CGC TCT TCC GAT CTC ACC AGA TAA CAG GTT CTT CTC TCT-3′ and reverse primer: 5′-TGG AGT TCA GAC GTG TGC TCT TCC GAT CTG TGT CTT TCA TTT CAC CAG GAA CA-3′ was used. Each sample was then given a unique 6-mer barcode (TruSeq primers, Illumina) by a nested PCR with primers targeting 5′- overhangs of the first PCR products. The barcoded *stra8* PCR products were mixed in equimolar ratios and the final denatured sequencing library was prepared at a concentration of 8 pM and spiked with 5% denatured phiX and sequenced on the MiSeq using MiSeq Reagent Kit v3 (600 cycle format). FastQ sequences were analyzed with CRISPResso2 (Clement et al., 2019) using the CRISPRessoPooled command in a customized Snakemake workflow (Köster and Rahmann, 2012). In brief, reads were filtered by retaining all reads with a minimum average Phred33 quality cutoff score of 20 and all bases with a minimum Phred33 score less than 20 were displayed as N. Trimming of Illumina adapters were enabled with the trimmomatic flag. In order to reduce sequencing errors occurring at the end of reads, paired-end reads were merged with FLASh and the preprocessed reads were then aligned by a global alignment algorithm aware of nuclease cut sites. An overview of the frequency and type of indels is shown in Supplementary Data 1.

### Gamete Quality

On September 26th of 2017, mature WT and *stra8* crispant males were stripped and the resulting sperm was then used for *in vitro* fertilization of WT eggs. Sperm samples from a total of five WT and six crispant males (of which three showed testes with gray areas) were used to individually fertilize ∼500–1000 eggs (average of ∼ 801 eggs per crossing for both genotypes) obtained from a single WT female. During the embryo incubation period, dead embryos were subsequently registered and removed daily. For all eleven different crosses, embryo survival was recorded post hatch on January 10th of 2018, as well as the number of embryos showing deformities.

### 11-KT Quantification by ELISA

The levels of 11-ketotestosterone (11-KT) were analyzed by ELISA (Cuisset et al., 1994) on extracted plasma samples, as previously described (Andersson et al., 2013). Acetylcholine esterase-labeled tracers and microplates pre-coated with monoclonal mouse anti-rabbit IgG were supplied by Cayman Chemicals. Anti-11-KT was a kind gift from David E. Kime (Sheffield University, United Kingdom).

### RNA Extraction and cDNA Preparation

A tissue piece of approximately 3 mm^3^ was homogenized in 400 μL of homogenization buffer and processed according to the Maxwell HT simplyRNA kit instructions (Promega) on a BioMek 4000 instrument (Beckton Dickinson). The quantity and purity of RNA samples were further assessed by spectrophotometry on a nanodrop ND-1000 instrument. cDNA was prepared by reverse transcription of 200 ng RNA using the SuperScript IV VILO Master Mix with ezDNase Enzyme according to the manufacturer’s recommendations (Thermo Fisher Scientific).

### Quantitative PCR (qPCR)

The primers and conditions for running the *vasa* and *ef1a* qPCR assays have previously been described (Wargelius et al., 2016). Primers for the remaining targets were designed using NCBI primer blast^1^ and their respective sequences are listed fully in Supplementary Table 1. The forward primer for the *stra8* gene was specifically designed to overlap the PAM site targeted by the exon-3 gRNA. A qPCR reaction was prepared according to the manufacturer descriptions to contain 800 nM of each forward and reverse primer in a 6 ul reaction containing a 1x concentration of the PowerUp SYBR Green master mix (Thermo Fisher Scientific). 2 μL of a 1/20 dilution of cDNA was added to the qPCR reaction and the reaction was subjected to thermocycling in a QuantStudio 5 Real-Time PCR system (Thermo Fisher Scientific) with an initial hold at 50°C for 2 min followed by an initial denaturation step at 95°C for 2 min. Thermocycling was conducted for 40 cycles using a denaturation step at 95°C for 1 s followed by a combined annealing and extension step at 60°C for 30 s. Data was processed at Thermo Fisher cloud using the relative quantification app.

### Phylogenetics

Stra8 protein sequences for phylogenetic analysis were retrieved from UniProt^2^. Phylogenetic trees and multiple alignment of Stra8 from several species were produced using the ETE toolkit (Huerta-Cepas et al., 2016) with the default standard_fastree workflow utilizing ClustalOmega and FastTree. Blocks of local alignment are depicted as gray blocks in the multiple alignments. Support values (0–1) depicted in the proximity to branches are calculated by the standard_fastree workflow as the result of 1000 bootstrap resamples.

### Apoptosis Detection by TUNEL

To determine the incidence of apoptosis, paraffin-embedded testis tissue was sectioned at 4 μm thickness and then subjected to deoxynucleotidyl transferase-mediated dUTP nick-end labeling (TUNEL). To this end, the sections were deparaffinized, rehydrated and then treated with permeabilization solution (20 μg/mL proteinase K in 10 mM Tris/HCl, pH 7.4) at 37°C for 30 min. Finally, slides were incubated with TUNEL reaction mixture (*In Situ* Cell Death Detection Kit, Fluorescein; Roche) in the dark at 37°C for 1 h. After washing twice in PBS, sections were counterstained with DAPI, mounted in Vectashield antifade mounting medium (Vector Laboratories) and imaged by confocal laser scanning microscopy (Zeiss LSM 700). Digital images were subjected to a quantification pipeline (see below). Negative and positive controls were included in each experimental set up (see Supplementary Figure 4B).

### Proliferation Analysis by Phospho-Histone H3 (pH3) Immunostaining

In order to assess germ cell proliferation, paraffin-embedded testis tissue was sectioned at 4 μm thickness and then subjected to an immunocytochemical procedure to detect the endogenous proliferation marker phospho-histone H3 (pH3). Paraffin sections were deparaffinized and rehydrated according to standard protocols and subjected to pH3 immunohistochemistry using a polyclonal rabbit anti-human pH3 (Ser10) antibody (Upstate^®^; Sigma-Aldrich) as previously described (Almeida et al., 2008), except that the primary antibody was detected by undiluted HRP-conjugated goat anti-rabbit IgG (Brightvision Immunologic).

### Image Quantification

Images from fluorescent stained sections (TUNEL) were quantified using a custom CellProfiler segmentation and quantification pipeline (Lamprecht et al., 2007). Images were first corrected for uneven illumination by the illumination correction function in CellProfiler. Thereafter a pixel classification was performed with Ilastik (Berg et al., 2019) to generate pixel probability maps for nuclei, background and TUNEL stained nuclei. The Watershed segmentation module in CellProfiler was then applied on the pixel probability maps. Objects identified by this module were then filtered by the filter and mask modules, before the final segmented, filtered and declumped objects were retained with the IdentifySecondaryObjects module. Five fields per individual and genotype were photographed at x20 magnification and the count of segmented nuclei and TUNEL labeled nuclei, as well as the percentage of TUNEL labeled nuclei, was then exported for analysis. Therefore, the total area investigated was the same for both WT and *stra8* crispant groups.

A separate CellProfiler pipeline was constructed for the analysis of pH3 3,3′-diaminobenzidine (DAB) labeled images. The percentage of positive stained areas was calculated using the segmented positively stained areas obtained from the Otsu two class thresholding algorithm of the IdentifyPrimaryObjects module in CellProfiler. Five different fields of view were quantified for each section. To obtain the single cell proliferation data, five representative fields for each individual were photographed. The images were then analyzed and the total number of pH3 positive intratubular single cells (i.e., type A undifferentiated spermatogonia and Sertoli cells) quantified. The two cell types have characteristic size and shapes of their nucleus, that can be used to identify them (pH3 staining is restricted to the nucleus, so that the cells can be identified reliably). The nuclei of type A undifferentiated spermatogonia are round and show the largest diameter (∼12 μm) of all germ cell nuclei. Sertoli cell nuclei are triangular, kidney or banana shaped but not round and quite a bit smaller (∼2 μm for the short dimension). This approach has been used previously to quantify single cell proliferation, including studies in Atlantic salmon (Crespo et al., 2019b).

### Testis Tissue Culture

A previously established primary testis tissue culture system developed for zebrafish (Leal et al., 2009a) was used, except that for salmon tissue incubations, the temperature was set to 14–15°C. Testis tissue was collected from immature males and incubated for 4 days in the absence or presence of 10 μM 4-diethylaminobenzaldehyde (DEAB, RA production inhibitor; Sigma-Aldrich) to investigate the effect of RA on gene expression in primary culture. At the end of the experiment, testis tissue was placed in RNAlater and stored at −80°C, until RNA extraction and qPCR analysis.

### Statistical Analysis

GraphPad Prism 8 package (GraphPad Software, Inc.) was used for statistical analysis. Significant differences between groups were identified using Student’s *t*-test (paired or unpaired, as appropriate) (^∗^*p* < 0.05; ^∗∗^*p* < 0.01; ^∗∗∗^*p* < 0.001). Data are represented as mean ± SEM.

## Results

### Salmon *stra8*: Phylogenetic Analysis and Testicular Expression During the Onset of Puberty

**FIGURE 1:**
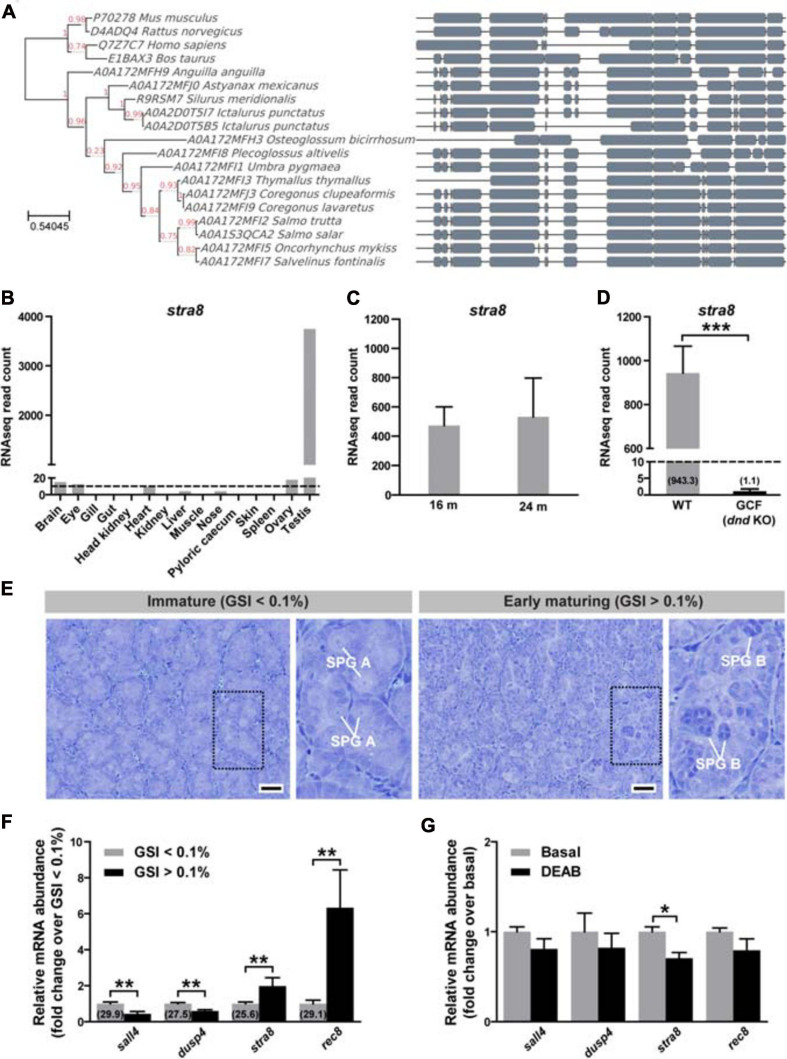
Phylogenetic tree of *stra8* and potential involvement of retinoic acid (RA) in male Atlantic salmon at puberty. **(A)** Phylogenetic tree produced from a multiple alignment of STRA8. Blocks of local alignment are shown as gray boxes along the multiple alignment. Support values (0–1) generated from 1000 bootstrap resamples are shown next to the branches in red. UniProtKB accession numbers are displayed in the tree next to the species name. **(B)** *stra8* expression levels in the different adult tissues used for making the reference annotation of the Atlantic salmon genome (GenBank GBRB00000000.1) (Lien et al., 2016). Results are expressed as RNAseq normalized read counts (*N* = 1). Dashed line indicates 10 RNAseq reads. **(C)** Testicular *stra8* expression in immature salmon males before and after smoltification. Results are shown as mean RNAseq normalized read counts ± SEM (*N* = 3–8) retrieved from GenBank PRJNA380580 data set (Kjærner-Semb et al., 2018) (16 m males) and own unpublished data (24 m males). **(D)** Testicular *stra8* expression in wild-type (WT) and germ cell-free (GCF) dead end (*dnd*) knockout salmon males, GenBank PRJNA550414 (Kleppe et al., 2020). Data are shown as mean RNAseq normalized read counts ± SEM (*N* = 3–4; ***p < 0.001). Numbers in brackets indicate average read counts for each group. Dashed line indicates 10 RNAseq reads. **(E)** Representative testis morphology of postsmolt males before (gonado-somatic index [GSI] < 0.1%) and after starting pubertal development (GSI > 0.1%). Insets show testis tissue magnified from the marked area (black dashed line), and representative type A (SPG A) and type B spermatogonia (SPG B) are labeled. Scale bar, 25 μm. **(F)** *In vivo* testicular expression of genes involved in spermatogonial differentiation and RA testicular function in postsmolt males before and after starting pubertal development. Numbers in brackets indicate *Cq* values obtained by qPCR analysis. Data are shown as mean fold change ± SEM (*N* = 8–9; **p < 0.01), and expressed relative to the control condition, which is set at 1. **(G)** Modulation of *sall4*, *dusp4*, *stra8* and *rec8* mRNA levels upon RA synthesis inhibition. Testicular fragments from immature males were cultured for 4 days at 14°C in the absence or presence of DEAB (10 μM). Data are shown as mean fold change ± SEM (*N* = 8–9; *p < 0.05), and expressed relative to the control condition, which is set at 1.

In the current ICSASG_V2 salmon reference genome the *stra8* gene is annotated as *stimulated by retinoic acid gene 8 protein homolog*. Within this reference there are four presumptive *stra8* genes LOC106599152, LOC106598674, LOC106609832, and LOC106590959. LOC106599152, LOC106598674, and LOC106609832 encode partial transcripts where two of these (LOC106599152 and LOC106598674) are located on unplaced short contigs, whereas LOC106609832 is placed on chromosome 7. The fourth, LOC106590959, encodes a full length 1478 base pairs (bp) *stra8* transcript, RefSeq accession number: XM_014182138.1. This transcript is located on an unplaced short contig and encodes a putative protein of 348 amino acids (XP_014037613.1), showing high sequence similarity to the *stra8* found in rainbow trout (*Oncorhynchus mykiss*) and brown trout (*Salmo trutta*). Phylogenetic analysis of the salmon *stra8* gene shows that it is clustered in the same branch as other salmonid species (Figure 1A).

Analysis of *stra8* transcript levels in deposited RNAseq data of the different adult tissues used in annotating the Atlantic salmon genome (GenBank GBRB00000000.1) (Lien et al., 2016) revealed high testicular expression (Figure 1B). Low read numbers were observed in the immature ovary, where most germ cells are primary previtellogenic oocytes arrested at the diplotene stage (data not shown). To obtain further information on *stra8* transcript levels in testis tissue, we examined *stra8* read numbers from three RNAseq data sets obtained from immature WT and germ cell-free mutant males (PRJNA380580 (Kjærner-Semb et al., 2018) and own unpublished data, 16 m and 24 m in Figure 1C, respectively; and PRJNA550414 (Kleppe et al., 2020), Figure 1D). Considerable expression was found in immature testis tissue from 16 or 24 months-old salmon (Figure 1C). No expression was found in testis tissue from germ cell-free *dead end* (*dnd*) knockout salmon confirming that the expression of *stra8* is limited to germ cells (Figure 1D).

To investigate whether *stra8* transcript levels change at the onset of pubertal development in male salmon, immature postsmolts (13–14 months old) were exposed to a maturation-inducing environmental regime (Fjelldal et al., 2011) for 16 days until the fish were sampled. In response to this regime, part of the fish had started pubertal development, as indicated by gonado-somatic index (GSI) levels above 0.1 and the presence of type B spermatogonia, respectively (Figure 1E). Elevated testicular transcript levels were recorded for *stra8* and also for *rec8*, a gene important for meiotic recombination but not directly regulated by STRA8 in mammals (Kojima et al., 2019). A brief summary of *stra8* and *rec8* gene functions, and of the other genes mentioned below, is given in Table 1. Transcript levels of *sall4* and *dusp4*, on the other hand, were halved in males, in which superallometric testis growth had started (GSI > 0.1%; Figure 1F). While *stra8* levels had increased further in testis tissue that started to mature, it was remarkable that *stra8* transcript levels were well above detection limit already in immature, prepubertal testis tissue, both before and after smoltification (16 m and 24 m, respectively; Figure 1C), that contained mainly type A undifferentiated spermatogonia (as shown in Figure 1E, left panel). Using immature testes in a primary tissue culture system, we found that adding the RA production inhibitor DEAB to the incubation medium down-regulated *stra8* transcript levels (Figure 1G). indicating that *stra8* expression also in salmon is stimulated by RA.

**TABLE 1:** Description of gene function and relevance to site of expression in the testis.

**Gene symbol**	**Expression in testis**	**STRA8 target (Kojima et al., 2019)**	**Gene function(s) in testis**	**References**
*Egr1*	Sertoli cells, testicular endothelial cells	No	Transcription factor that stimulates expression of *Dmrt1* in Sertoli cells. Stimulates SSC self-renewal by increasing GDNF	Lei and Heckert (2002), Bhang et al. (2018)
*Dusp4*	Undifferentiated SPG	No	Inhibits JNK-mediated stimulation of SSC self-renewal	Chan et al. (2017)
*Lin28a*	Undifferentiated SPG	No	Promotes self-renewal proliferation of SSCs. Marker for undifferentiated type A spermatogonia	Chakraborty et al. (2014), Shami et al. (2020)
*Ret*	Undifferentiated SPG	No	Regulates GDNF-dependent SSC self-renewal	Naughton et al. (2006)
*Nanos2/nanos2*	Undifferentiated SPG, type A SPG	No	RNA binding protein that regulates homeostasis of murine SSCs. Potential SSC marker in fish	Beer and Draper (2013), Bellaiche et al. (2014), Zhou et al. (2015)
*Upp1*	Undifferentiated SPG	No	Potential progenitor cell marker	La et al. (2018)
*Pou5f1*	Undifferentiated SPG, early differentiating SPG	No	Its down-regulated expression in early differentiating spermatogonia is necessary for the progression of spermatogenesis	Zheng et al. (2016)
*Dmrt1*	SPG, early SPC, Sertoli cells	* -	Known to repress *Stra8*	Raymond et al. (2000), Matson et al. (2010), Webster et al. (2017), Zhang and Zarkower (2017), Grive et al. (2019), Kojima et al. (2019)
*Sall4*	SPG	No -	RA inducible, promoting spermatogonial differentiation by sequestering PLZF and regulating the expression of KIT	Gely-Pernot et al. (2015), Green et al. (2018), Grive et al. (2019), Kojima et al. (2019)
*Ccnd2*	SPG	No	Possible role in supporting differentiation of SPG	Beumer et al. (2000), Grive et al. (2019)
*piwil1*	SPG	No	Endoribonuclease that in conjunction with piRNAs represses the activity of transposable elements in order to protect germline integrity	Houwing et al. (2007), Thomson and Lin (2009)
*Dazl*	SPG, early SPC	* +	RNA binding protein essential for gametogenesis. Promotes a more robust translation of a large fraction of mRNAs in spermatogonia, facilitating the proliferation and differentiation of progenitor spermatogonia	Zagore et al. (2018), Grive et al. (2019), Kojima et al. (2019), Mikedis et al. (2020)
*Kit*	Differentiating SPG, Leydig cells	No	Tyrosine kinase receptor for the ligand stem cell factor. Supporting proliferation of differentiated SPG	Rossi et al. (2000), Green et al. (2018), Grive et al. (2019)
*Dmc1*	Leptotene to zygotene SPC	* +	RecA homolog that is specifically expressed during meiosis. Stimulated by STRA8 and mediates crossover in the meiotic phase	Yoshida et al. (1998), Koubova et al. (2014), Kojima et al. (2019), Paiano et al. (2020)
*Prdm9*	SPG, early SPC	* +	Zinc finger containing histone H3K4 trimethylase that is expressed in early meiosis and regulates mammalian recombination hotspots	Parvanov et al. (2010), Grey et al. (2018), Ma et al. (2018), Grive et al. (2019), Kojima et al. (2019), Paiano et al. (2020)
*Ccna1*	SPC	No	Loss of *Ccna1* in mice results in disruption of spermatogenesis and male sterility due to cell arrest and apoptosis in the late diplotene stage of the meiotic cell cycle	Wolgemuth (2011)
*Sycp3*	SPC	* +	Structural component of the synaptonemal complex involved in synapsis, recombination and segregation of meiotic chromosomes. Required for normal meiosis during spermatogenesis	Yuan et al. (2000), Green et al. (2018), Grive et al. (2019), Kojima et al. (2019)
*Spo11*	SPC	*	Catalyzes the formation of DNA double strand breaks during meiosis	Keeney (2008), Green et al. (2018), Kojima et al. (2019)
*Rec8*	SPC	No -	Part of the cohesin complex that mediates sister chromatid cohesion during meiosis	Parisi et al. (1999), Kojima et al. (2019)
*Sox30*	Mid pachytene SPC, peak of expression in round elongated SPT	No	Transcription factor essential for post meiotic spermiogenesis phase	Feng et al. (2017)

(*) Proximal promoters found to be bound by STRA8 by chromatin immunoprecipitation in preleptotene spermatocytes; (−) down-regulated in *Stra8*^+/–^ vs. *Stra8*^–/–^ preleptotene spermatocytes; (+) up-regulated in *Stra8*^+/–^ vs. *Stra8*^–/–^ preleptotene spermatocytes (Kojima et al., 2019). SPG, spermatogonia. SPC, spermatocytes. SPT, spermatids.

### Genetic Ablation of *stra8* in Salmon Transiently Disturbed Spermatogenesis

**FIGURE 2:**
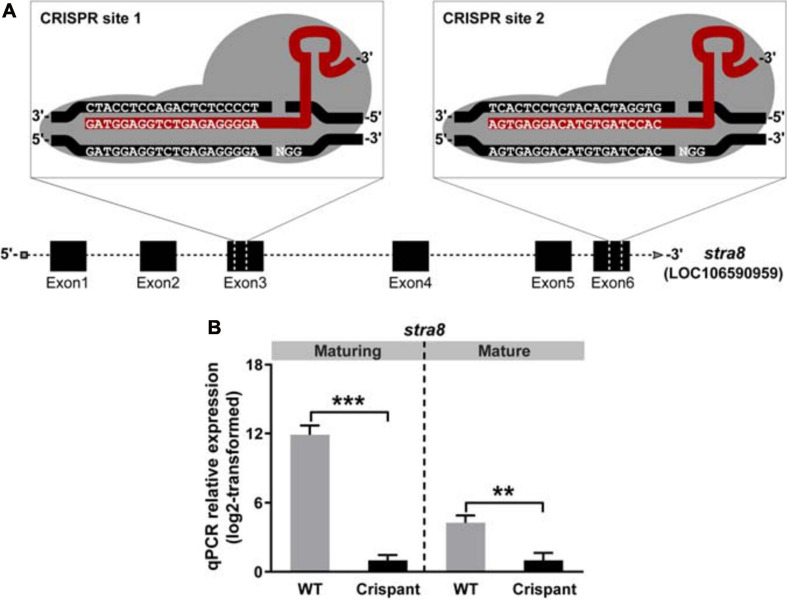
Efficient disruption of salmon *stra8* by CRISPR/Cas9 and *stra8* germ cell-specific expression. **(A)** Design of guide RNA target sites on exon 3 and exon 6 targeting the *stra8* gene. **(B)** CRISPR/Cas9-mediated decrease of WT *stra8* transcript levels in both maturing and mature Atlantic salmon crispant males. Data are shown as mean relative expression ± SEM (*N* = 7–10; **p < 0.01; ***p < 0.001). WT, wild-type.

We generated *stra8* crispants using CRISPR/Cas9. Guide RNAs (gRNAs) were designed to target exons 3 and 6 (Figure 2A). MiSeq analysis of fin clips of *stra8* crispant fish, revealed that the gRNA targeting exon 3 induced mutations at the targeted locus at a high efficiency, whereas the second gRNA targeting exon 6 was non-functional. An overview of the frequency and type of indels can be found for each individual in Supplementary Data 1. qPCR of testis from the mutants showed a 4- or 12-log_2_ fold reduction of *stra8* mRNA levels in the testis compared to WT siblings, in mature or maturing males during the rapid testicular growth phase (Figure 2B).

**FIGURE 3:**
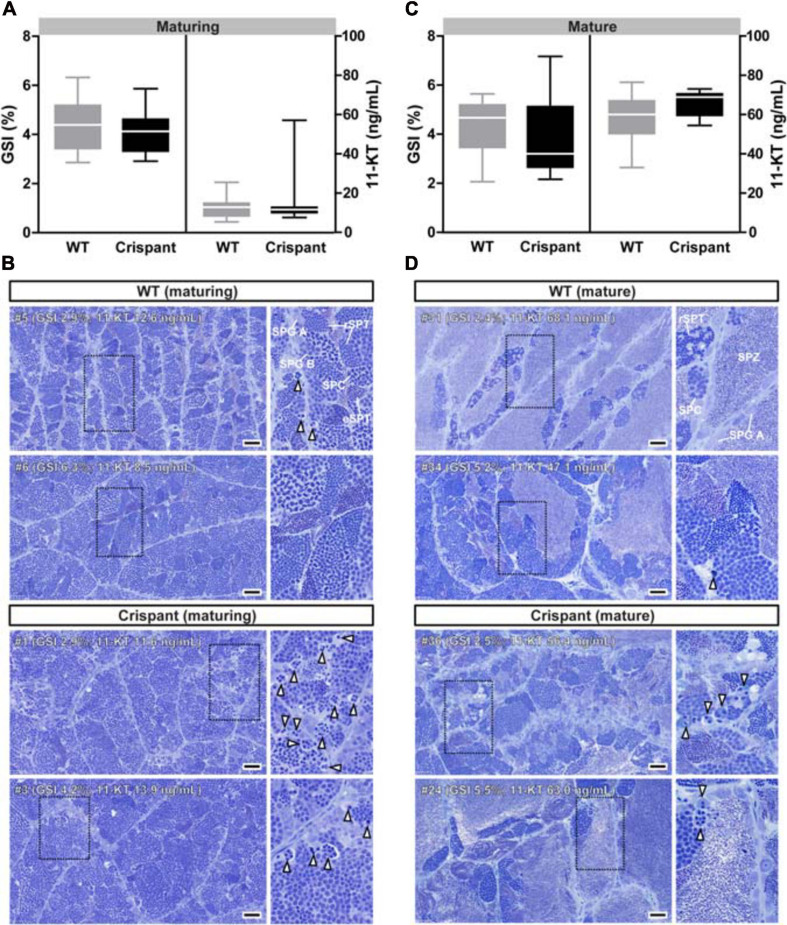
Evaluation of gonadal tissue and plasma androgen levels of WT and *stra8* crispant salmon males. **(A,C)** Gonado-somatic indices (GSI) and plasma androgen (11-ketotestosterone; 11-KT) levels in WT and *stra8* crispant males at samplings from May (maturing) and September 2017 (mature). **(B,D)** Histological images of maturing **(B)** and mature **(D)** Atlantic salmon testes obtained from WT and *stra8* crispant fish. Insets show testis tissue magnified from the marked area (black dashed line). White arrowheads indicate apoptotic germ cells. Representative germ cell stages are shown in the upper right panels: type A spermatogonia (SPG A), type B spermatogonia (SPG B), spermatocytes (SPC), round spermatids (rSPT), elongated spermatids (eSPT) and spermatozoa (SPZ). WT, wild-type; Crispant, *stra8* crispant. Scale bar, 50 μm.

The relative testis weight remained unaffected in maturing and mature *stra8* crispants (GSI; Figure 3A). However, analyzing histological sections from maturing testes revealed clear effects on spermatogenesis in *stra8* crispants, such as numerous apoptotic nuclei (white arrowheads in Figure 3B) and an irregular organization of spermatogenic cysts (Supplementary Figure 1A). Moreover, some *stra8* crispant testes (e.g., #8 and #11; Supplementary Figure 1A) showed an accumulation of type B spermatogonia and few meiotic and postmeiotic cells, which differed from the appearance of testes sections from WT individuals. Neither plasma 11-KT (main androgen in fish) nor expression levels of steroidogenesis-related genes were affected in *stra8* crispant males compared to WT siblings (Figure 3A and Supplementary Figure 2, respectively). Remarkably, 4 months later spermatogenesis had largely recovered in *stra8* crispants. Many tubuli contained large numbers of spermatozoa. The spermatogenic cysts still present had progressed in development and mainly contained spermatocytes and spermatids, while the different generations of spermatogonia were still present but no longer in a prominent manner (Figure 3D). As observed in maturing males, GSI values and 11-KT plasma concentrations in mature *stra8* crispants were similar to those in WT controls (Figure 3C). Despite the apparent spermatogenic recovery, some tubuli in mature *stra8* crispant testes still contained several apoptotic cells (e.g., male #36 in Figure 3D), in particular in areas still comparatively rich in earlier stages of spermatogenesis with many type B spermatogonia and spermatocytes that macroscopically showed a gray coloration otherwise seen also in maturing testes (males #25, #30 and #32 in Supplementary Figures 3A,B), while the complete testis was white-colored and contained lumina with many spermatozoa in WT controls (Supplementary Figure 3B, male #29) and most – 7 out of 10 – *stra8* crispants (Supplementary Figure 3B; e.g., male #28), but also in the white areas of the three *stra8* crispant testes showing gray areas.

Experiments on gamete quality (Supplementary Figure 3C) showed that sperm quality was indistinguishable for both genotypes, irrespectively of the gonad coloration of the crispants, considering that three of the six crispant sperm samples were collected from the males showing gray testis tissue parts. Fertilization capacity, embryo survival and percentage of embryos showing deformities, recorded at hatching stage, were all similar to the values observed in WT controls.

**FIGURE 4:**
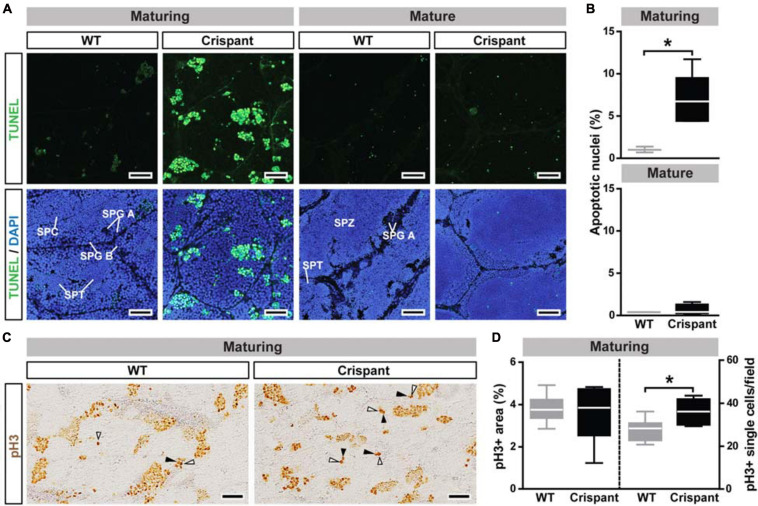
Germ cell apoptosis and proliferation in WT and *stra8* crispant salmon testis tissue. **(A,B)** Detection **(A)** and quantification **(B)** of germ cell apoptosis/DNA damage by TUNEL analysis. TUNEL + cells/cysts are shown in green and DAPI counterstain in blue. Representative germ cell stages are labeled: type A spermatogonia (SPG A), type B spermatogonia (SPG B), spermatocytes (SPC), spermatids (SPT) and spermatozoa (SPZ). Scale bar, 30 μm. Quantification results are shown as mean ± SEM (*N* = 3–5; *p < 0.05). **(C,D)** Detection **(C)** and quantification **(D)** of cell proliferation by pH3 analysis. White and black arrowheads indicate pH3 + type A undifferentiated and Sertoli cells, respectively (examples of proliferating cells are shown at higher magnification in Supplementary Figure 4C). Scale bar, 50 μm. WT, wild-type; Crispant, *stra8* crispant.

TUNEL analysis confirmed a sevenfold increased frequency of germ cell death by apoptosis in maturing, but not in mature, *stra8* crispants (Figures 4A,B). In maturing mutants, DNA fragmentation was mainly observed in type B spermatogonia and spermatocytes, as identified by the shape and size of their DAPI-stained nuclei (Supplementary Figure 4A). Since GSI were not different despite elevated germ cell loss to apoptosis, we hypothesized that mutant testes may show a higher proliferation activity. While no differences were found in overall germ cell proliferation (Figures 4C,D left panel), single cell proliferation activity was significantly elevated in mutants (Figure 4C arrowheads and Figure 4D right panel). This increased activity involved single type A_und_ spermatogonia and associated Sertoli cells (white and black arrowheads, respectively, on Figure 4C and Supplementary Figure 4C).

### Analysis of Cell Stage Spermatogenic and Meiotic Markers in *stra8* Crispants

To evaluate further potential effects in *stra8* crispants on the three main phases of spermatogenesis (mitotic, meiotic, and spermiogenic phases), we quantified the expression levels of several genes expressed in germ cells, and of some genes expressed also or only in somatic cells (e.g., *dmrt1* and *egr1*, respectively; see Table 1). Moreover, some of the genes analyzed (indicated by an asterisk in Table 1) are known direct downstream targets of STRA8 in mammals.

**FIGURE 5:**
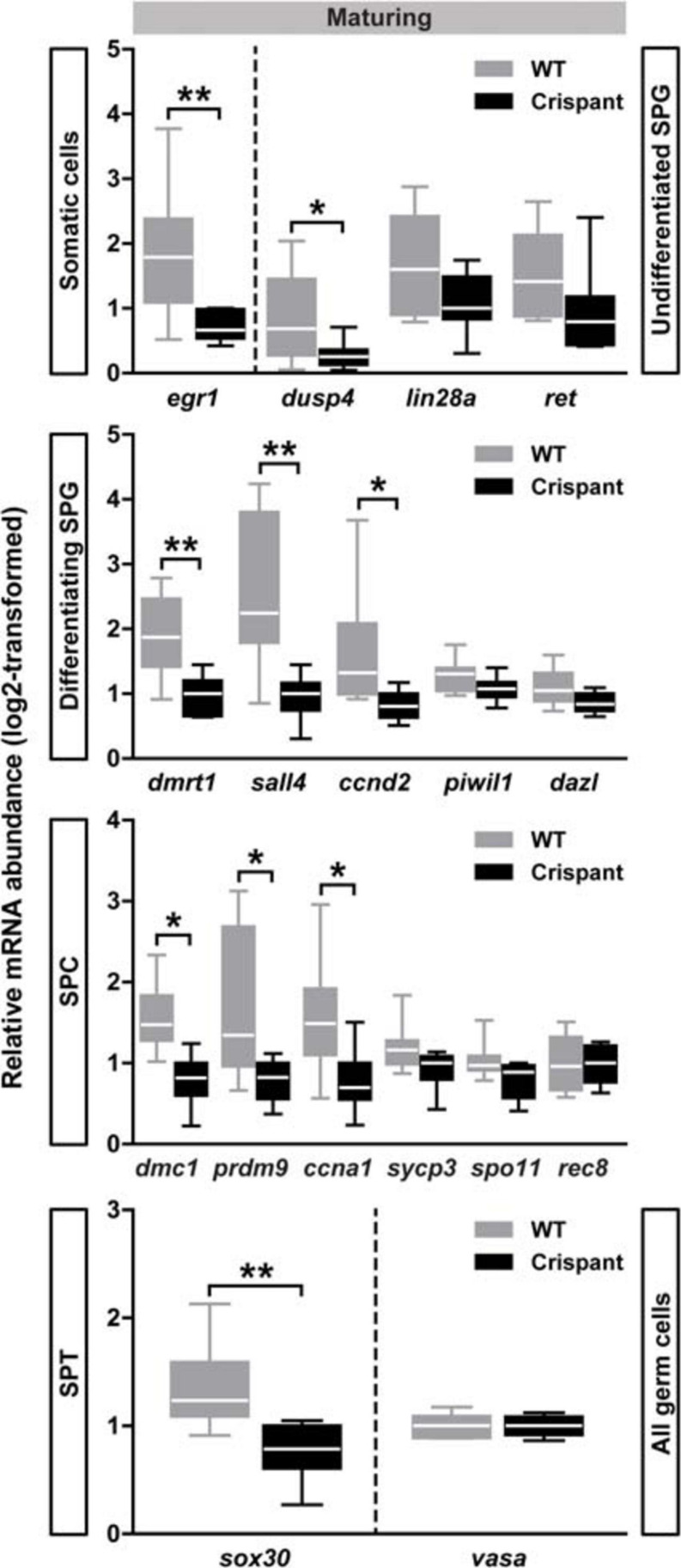
Testicular gene expression in maturing WT and *stra8* crispant fish. Transcript levels of selected markers for germ cells at different stages of development are shown as mean ± SEM (*N* = 7–10; *p < 0.05, **p < 0.01), and expressed relative to *ef1a* expression. WT, wild-type; Crispant, *stra8* crispant; SPG, spermatogonia; SPC, spermatocytes; SPT, spermatids.

Considering the early generations of spermatogonia, several genes showed similar expression levels in maturing WT and *stra8* crispants (e.g., *lin28a, ret, nanos2, upp1, pou5f1*; see Undifferentiated SPG in Figure 5 and Table 1), suggesting that the spermatogonial stem cells (SSCs) and progenitor generations among the type A spermatogonia were not drastically affected by the loss of *stra8*. This is in line with our morphological observations, since also in *stra8* crispants the SSCs were able to self-renew and to produce differentiating spermatogonia, of which many completed the spermatogenic process. However, we did record significantly decreased transcript levels in maturing *stra8* crispant testes of two genes (*dusp4* and *egr1*; Figure 5), which encode proteins that in mammals are involved in the regulation of SSC proliferation and differentiation (Chan et al., 2017; Bhang et al., 2018). Considering transcripts expressed in differentiating spermatogonia, no significant differences were observed regarding *dazl, piwil1* (Figure 5), or *kit* (Table 1), while three transcripts associated with differentiating spermatogonia and their mitotic expansion (*ccnd2*, *dmrt1* and *sall4*) were significantly reduced in crispants (Figure 5). Also with respect to meiosis markers, we found genes that did (*dmc1*, *prdm9*, *ccna1*; Figure 5) or did not show (*rec8*, *sycp3*, *spo11*; Figure 5) significant expression changes in crispants. The only spermatid marker examined (*sox30*) showed reduced transcript levels in crispants, while no differences were found for the general germ cell marker *vasa* (Figure 5). The expression levels of growth factors known to regulate the differentiation and proliferation behavior of spermatogonia did not change in maturing *stra8* crispants (Supplementary Figure 2). None of the genes investigated by qPCR in the present study showed significant changes in testis tissue of mature *stra8* crispants (data not shown).

Taken together, our observations show that the expression of *stra8* was clearly detectable already in immature testes, confined to germ cells, up-regulated during the onset of puberty and decreased when blocking RA production. In maturing *stra8* crispants, the expression of some genes important for the mitotic or meiotic phases of spermatogenesis was reduced, while several other transcripts remained unchanged. Reduced *stra8* levels were associated with clear defects during spermatogenesis when the maturing salmon testis progressed through its rapid growth phase. However, these defects had been compensated when the males approached the fully mature stage of development.

## Discussion

### Phylogeny and Tissue Specificity of Salmon *stra8*

Our first aim was to identify *stra8* in Atlantic salmon. While *stra8* localized to a contig too short for synteny analysis, the phylogenetic analysis based on 14 fish species with an annotated *stra8* gene (Figure 1A) indicated that the gene found in salmon was a *stra8* ortholog. This annotation is supported by the germ cell-specific expression of this gene also in salmon (Kleppe et al., 2020) (Figure 1D), as previously reported in other fish species (Dong et al., 2013; Li et al., 2016; Pasquier et al., 2016). However, in contrast to the observation in Southern catfish (Dong et al., 2013), *stra8* transcripts were barely detectable in immature salmon ovaries. It is possible that higher *stra8* transcript levels can be recorded also in salmon when oogonia enter meiosis, but we did not collect samples covering the period that this (earlier) stage of female germ cell development is relatively prominent in the juvenile ovary. In any case, it seems that *stra8* expression is not required to safeguard the existence of previtellogenic primary oocytes that have, as usual for oocytes until resumption of meiosis when approaching ovulation, arrested in diplonema.

### *stra8* in Salmon Testis

We found considerable amounts of *stra8* transcripts in immature testes not containing differentiating spermatogonia or spermatocytes (Figures 1C,F,G). In contrast, *Stra8* transcripts are not abundant in mice until they reach puberty, when RA strongly increased *Stra8* levels in differentiating spermatogonia although low levels were already present in undifferentiated spermatogonia (Zhou et al., 2008). There is little information on the functional context of the expression of *stra8* in immature salmon testis with spermatogenic tubules containing only undifferentiated spermatogonia and Sertoli cells. Early *stra8* expression may prepare immature germ cells for an upcoming activation of spermatogonial differentiation/entry into meiosis, while the transcript may not be used yet, perhaps being idle while associated with RNA binding proteins. Unfortunately, we have no information on Stra8 protein levels in the testis of salmon or other fish species. Considering that *stra8* transcript levels were reduced after exposing immature testis tissue to a compound preventing RA biosynthesis, or increased in testis entering puberty following photoperiod stimulation (Figures 1F,G), we assume that locally produced RA increased *stra8* expression in the immature salmon testis to support the transition to differentiating spermatogonia (Zhou et al., 2008). Respective data is not available for testis tissue of other fish species. However, in Southern catfish ovaries, experimentally reduced or increased RA levels, also reduced or increased, respectively, the expression of *stra8* (Li et al., 2016). These observations suggest that fish share RA-mediated regulation of *stra8* gene expression with higher vertebrates. In fish lacking the *stra8* gene, modulating gonadal RA levels still was relevant for spermatogenesis (Feng et al., 2015; Crespo et al., 2019a), demonstrating the activity of *stra8*-independent, RA-modulated pathways to promote the mitotic phase of spermatogenesis.

### Maturing Testis of *stra8* Crispants Are Characterized by Increased Apoptosis and Single Cell Proliferation

Loss of *Stra8* in mice leads to sterility with an arrest of male germ cell differentiation at the spermatogonial stage and a hypoplastic testis (Anderson et al., 2008). In contrast to this finding in mice, testis weight or GSI did not differ in WT and *stra8* crispant salmon, which also produced meiotic and postmeiotic germ cells. However, during the rapid growth phase of the maturing testis characterized by containing many type B spermatogonia, spermatocytes and spermatids, *stra8* crispants displayed disorganized spermatogenic tubuli with a clearly higher incidence of germ cell apoptosis in comparison to WT controls, which mainly occurred in type B spermatogonia and spermatocytes. It is therefore conceivable that one of the functions of salmon Stra8 is to reduce germ cell apoptosis. Germ cell apoptosis is a common phenomenon in normal vertebrate spermatogenesis (Shaha et al., 2010). In zebrafish, for example, ∼40% of the germ cells are lost before they reach the stage of mature spermatozoa in WT males, and interestingly, the losses mainly occurred among type B spermatogonia and spermatocytes (Leal et al., 2009b). Also in Atlantic salmon, ongoing work in our group indicates that the main cell loss in WT testes occurs among type B spermatogonia and spermatocytes (unpublished results). While apoptotic germ cell loss is normal, the degree to which it occurred in maturing *stra8* crispants was remarkably higher than in maturing WT controls. We have no information on the molecular mechanism(s) used by Stra8 to prevent germ cell apoptosis in salmon. However, recent work in mice showed that STRA8 inhibited germ cell caspases via the AKT pathway to exert an anti-apoptotic effect (Shen et al., 2018). In case in salmon a similar mechanism should be active, it may explain the higher apoptotic index observed in crispants, which mainly affected the germ cell stages also more susceptible to apoptosis in WT males.

Normal GSI values in maturing testes suggest that the apoptosis-related loss of germ cells somehow was compensated, for example by changes in proliferation activity. While we did not find differences in the overall testicular proliferation activity, we detected an increase in the single cell proliferation activity in *stra8* crispants, involving type A_und_ spermatogonia and associated Sertoli cells. In the cystic type of spermatogenesis typically seen in fish, Sertoli cells envelope and accompany a single germ cell clone during spermatogenesis (Schulz et al., 2010), thus forming the functional unit of fish spermatogenesis, known as spermatogenic cyst. Since new cysts are formed when a type A_und_ spermatogonium derived from a self-renewal division associated with a Sertoli cell, we assume that the elevated level of single cell proliferation of specifically these two cell types increased the production of spermatogenic cysts in maturing *stra8* crispants. Producing more cysts may allow the crispants to reach normal GSI despite losing many more germ cells to apoptosis than WT controls. In salmon, there is no information on possible drivers of Sertoli cell and type A_und_ proliferation. However, in both zebrafish (Nóbrega et al., 2015; Safian et al., 2018) and mice (reviewed by Oduwole et al., 2018), Fsh-mediated effects are very important for the proliferation of these cells. It will be interesting to investigate in future studies if, for example, *stra8*-deficient testis responds differently to Fsh than WT testis. It is not clear how the increased apoptotic loss of germ cells is perceived and then communicated to the (as yet also unknown) regulatory system controlling cyst production in *stra8* crispants. However, once the signaling in the newly formed cyst changes to induce differentiation, resulting in a series of mitotic divisions that increases germ cell number geometrically, elevated cyst production seems a feasible route to compensate the apoptotic germ cell loss.

### Expressional Changes Associated With the Mitotic Phase

As discussed above, the response of the immature testis to the loss of *stra8* may be linked to an expansion of the stem cell reservoir, eventually resulting in additional spermatogenic cysts. Proteins encoded by *gsdf, igf3, insl3* or *amh* stimulate or inhibit, respectively, spermatogonial proliferation (Sawatari et al., 2007; Assis et al., 2016; Morais et al., 2017). However, none of them changed (Supplementary Figure 2). Considering that three of these growth factors respond to endocrine regulation by Fsh and/or androgens (Sambroni et al., 2013; Melo et al., 2015; Nóbrega et al., 2015; Crespo et al., 2016), and that circulating androgen levels in *stra8* crispants were similar to those in WT controls (Figures 3A,C), it seems unlikely that mutant males differed largely from controls regarding the gonadotropin and/or androgen mediated regulation of spermatogenesis. However, local testicular signaling systems may well have responded to the loss of *stra8*. In this regard, the decreased *dusp4* transcript levels may be relevant, considering that in mice, the protein phosphatase DUSP4 inhibits JNK-mediated stimulation of SSC self-renewal (Chan et al., 2017). Assuming that a similar mechanism is active in the salmon testis, decreased *dusp4* transcript levels may contribute to the observed increase in proliferation of single type A_und_ spermatogonia that feeds into the production of additional spermatogenic cysts. However, other transcripts associated with SSC or progenitor spermatogonia remained unaffected in *stra8* crispants (e.g., *ret*, *lin28a*, *pou5f* and *upp1*). Taken together, there is little molecular and no morphological evidence for the notion that SSCs or progenitor spermatogonia functions are compromised in *stra8* crispants. In contrast, our observations rather indicate an elevated activity, leading to an increased production of spermatogenic cysts.

In transcripts encoding proteins exerting functions in either differentiating spermatogonia (*ccnd2*, *sall4, dmrt1, piwil1* and *dazl*) or in Sertoli cells (*dmrt1* and *egr1*), decreased transcript levels were observed for *ccnd2*, *sall4, dmrt1* and *egr1*, while *piwil1* and *dazl* remained unchanged in maturing *stra8* crispants. These observations suggest that the transition from undifferentiated to differentiating spermatogonia was partially compromised in *stra8* crispants. Studies in mice linked CCND2 to spermatogonial differentiation (Beumer et al., 2000). Also in mice, *Sall4* gene expression is, as *Stra8*, directly induced by RA, upon which SALL4 sequesters the transcriptional inhibitor ZBTB16 (a.k.a. PLZF), eventually promoting spermatogonial differentiation (Gely-Pernot et al., 2015). Lower levels of the two germ cell-specific transcripts *sall4* and *ccnd2* in *stra8* crispants therefore suggest that the transition from undifferentiated to differentiating spermatogonia was partially compromised. The transcription factor *dmrt1* is expressed in both Sertoli cells and spermatogonia in mice and zebrafish and germ cell-specific deletion of *Dmrt1* in mice caused spermatogonia to precociously exit mitotic cell cycling and enter meiosis (Matson et al., 2010). In zebrafish, the generalized loss of *dmrt1* also resulted in a hypoplastic testis, but in contrast to mice, the small number of spermatogonia are also unable to enter meiosis and eventually, testis tissue becomes devoid of germ cells (Webster et al., 2017), suggesting that spermatogonial stem cell maintenance was dysregulated as well. The more severe phenotype in zebrafish may reflect the loss of *dmrt1* function also from Sertoli cells, since the combination of inability to enter meiosis and loss of stem cells was also described in mice with a generalized loss of *Dmrt1* (Raymond et al., 2000). Interestingly the phenotype also included overproliferation of Sertoli cells, reminiscent of what we see in *stra8* crispants. Possibly, Stra8 can stimulate Sertoli cell proliferation and cyst formation through a yet unknown pathway that may involve germ-to-Sertoli cell signaling, since *stra8* crispants also showed reduced levels of *egr1*, encoding a transcription factor that stimulates *Dmrt1* expression in murine Sertoli cells (Lei and Heckert, 2002). There is no experimental evidence for germ-to-soma signaling in the salmon testis so far, but in mice germ cells use Notch ligands to stimulate Sertoli cell Notch receptors (Garcia et al., 2017). Overall, more work is required in salmon to clarify the mechanisms connecting *stra8* to *sall4, dmrt1* and *egr1*, considering that in mice, SALL4 functions in parallel to and independent of STRA8 while DMRT1 is up-stream of STRA8 in germ cells (Matson et al., 2010; Gely-Pernot et al., 2015; Zhang and Zarkower, 2017). The fact that *piwil1* was not modulated in crispants may reflect the constitutive functions of Piwi proteins for germ cell genome stability (Thomson and Lin, 2009). Also *dazl* transcript levels were stable. This gene encodes an RNA binding protein that promotes the more robust translation of a large fraction (∼30%) of transcripts in spermatogonia, thereby facilitating the differentiating proliferation of progenitor spermatogonia in mice (Zagore et al., 2018; Mikedis et al., 2020). Our observations on *piwil1* and *dazl* thus are in line with the notion that their stable expression contributes to the requirements for germ cell survival and their developmental transition into differentiating spermatogonia that is still taking place in *stra8* crispants, although partially compromised.

### Expressional Changes Associated With Meiosis and Spermiogenesis

In mice, STRA8-deficient spermatocytes initiate, but fail to complete meiosis, so that mutant germ cells still express genes needed for the initiation of meiosis, such as *Sycp3*, *Spo11* and *Rec8* (Mark et al., 2008). Likewise, we observed no differences in the expression of these genes between control males and *stra8* crispants during the rapid growth phase of the maturing testis (Figure 5), while other meiosis-associated factors including *ccna1, prdm9* and *dmc1* were reduced in mutants. In mice, loss of *Ccna1* disrupts spermatogenesis by arresting the first meiotic cell cycle in the late diplotene stage, resulting in the loss of these spermatocytes to apoptosis (Wolgemuth, 2011). It is unknown if *stra8* controls *ccna1* expression in salmon, but the increased apoptosis among spermatocytes in *stra8* crispants may indicate that reduced *ccna1* and elevated apoptosis are linked in the salmon testis as well. PRDM9 is a zinc finger containing histone H3K4 trimethylase that is expressed in early meiosis and *Prdm9*-deficient mice are sterile (Parvanov et al., 2010). STRA8 directly modulated *Prdm9* gene expression in mice (Ma et al., 2018; Kojima et al., 2019). PRDM9 interacts with SPO11, initiating DNA double-strand breaks (DSBs) that are repaired by the recombinases DMC1 and RAD51 in the meiosis crossover process (Parvanov et al., 2010; Grey et al., 2018; Paiano et al., 2020). A shortage of either Prdm9 or Dmc1 protein in salmon *stra8* crispants may have resulted in DSB repair problems and hence elevated apoptosis among spermatocytes. Based on a *stra8* mouse mutant data (Koubova et al., 2014; Kojima et al., 2019), finding reduced *dmc1* transcript levels in *stra8* crispant salmon was not surprising. Finally, *Sox30* in mice is expressed at the end of meiosis and during spermiogenesis (Bai et al., 2018). The reduced *sox30* transcript levels in *stra8* crispants may reflect a smaller number of spermatids resulting in a cumulative manner from the several effects discussed above during the mitotic and meiotic phases. Overall, our data suggests that also in the Atlantic salmon, *stra8* supported the completion of meiosis, but different from mammals, is not required for meiosis.

### Phenotype and Compensation in the Mature *stra8* Crispant Testis

Eventually, *stra8* crispant testes reached the mature stage and produced functional sperm (Figure 3D and Supplementary Figure 3), which could fertilize eggs at a similar rate as control sperm (Supplementary Figure 3C). These results further indicate that alternative pathways to complete meiosis exist also in fish species that do have a *stra8* gene, pathways possibly similar to those used in, for example, zebrafish, medaka and tilapia (Feng et al., 2015; Adolfi et al., 2016; Crespo et al., 2019a), which miss a *stra8* gene in their genome. Nevertheless, the removal of *stra8* did have noticeable consequences, such as increased apoptotic loss of germ cells and increased single germ and Sertoli cell proliferation. The latter probably reflects the production of additional spermatogenic cysts, which can be understood as compensatory response to level out the increased loss of germ cells to apoptosis. This compensation took place during the growth phase of the maturing testis. When GSI values reach their maximum during the annual reproductive cycle of Atlantic salmon, this indicates that the production of new spermatogenic cysts stops while existing cysts continue to differentiate so that the testis becomes filled with mature sperm; single undifferentiated spermatogonia are the only other germ cell type present and remain quiescent until they resume activity at the beginning of the next annual cycle (Schulz et al., 2010). The mature status of both WT controls and *stra8* crispants is indicated by the large number of mature sperm and androgen plasma levels of 60–80 ng/mL (Figure 3). Therefore, it was not surprising that neither cyst production nor apoptosis were different when comparing the two mature groups. After all, the processes were largely completed and the cell types involved had reached postmeiotic stages of development, or were lost to apoptosis, and hence no longer present in the mature testis. Also, while RA deficiency completely blocks spermatogonial differentiation in mammals, blocking RA production reduced, but did not block, sperm production/fertility in zebrafish (Pradhan and Olsson, 2015). These observations suggest that also *stra8*-independent but RA-regulated processes are not strictly required for spermatogenesis in fish. It appears that in teleost fish, signaling pathways regulating spermatogenesis operate in parallel, such that failure of a single pathway can be compensated at least in part in many cases. In mammals, on the other hand, some of these pathways apparently are organized in a sequential manner, such that when one of the elements operating in sequence fails, the spermatogenic process is blocked at this then crucial bottleneck with no option for alternative routes. In this context it is relevant to consider that (in context with the long generation time in salmon) we carried out our work with F0 crispants. The mosaicism of the F0 crispants and hence the presence of low amounts of WT in frame mutations may have provided, if any, only minor rescue effects in the crispants, considering the less than 1% WT sequence found in any of the crispants (see Supplementary Data 1).

## Data Availability Statement

The datasets presented in this study can be found in online repositories. The names of the repository/repositories and accession number(s) can be found below: https://www.ncbi.nlm.nih.gov/, PRJNA380580; https://www.ncbi.nlm.nih.gov/, PRJNA550414; and https://www.ncbi.nlm.nih.gov/genbank/, GBRB00000000.1.

## Ethics Statement

The animal study was reviewed and approved by the Norwegian Animal Research Authority (NARA, permit number 5741) and the use of these experimental animals was in accordance with the Norwegian Animal Welfare Act.

## Author Contributions

KOS, DC, LK, EA, RBE, BN, and AW performed the experiments. KOS, DC, AW, and RWS analyzed and contributed to the interpretation of the results. AW, RWS, TJH, PGF, and RBE conceived the project, secured funding, and provided the supervision. KOS, DC, RWS, and AW wrote the manuscript. All authors contributed to the article and approved the submitted version.

## Conflict of Interest

The authors declare that the research was conducted in the absence of any commercial or financial relationships that could be construed as a potential conflict of interest.

## Footnotes

1 https://www.ncbi.nlm.nih.gov/tools/primer-blast/

2 https://www.uniprot.org
