# Supplementary material for: Loss of stra8 Increases Germ Cell Apoptosis but Is Still Compatible With Sperm Production in Atlantic Salmon (Salmo salar)
Source: Front Cell Dev Biol. 2021 Apr 16;9:657192. doi: 10.3389/fcell.2021.657192 (PMC8087537; doi:10.3389/fcell.2021.657192)
Supplement: Supplementary file 2 [file Table_2.DOCX]

Supplementary Material

# Supplementary Data

**Supplementary Data 1. MiSeq analysis: overview of the frequency and type of indels for each *stra8* crispant individual.**

Attached as a separate file.

# Supplementary Figures and Tables

## Supplementary Figures


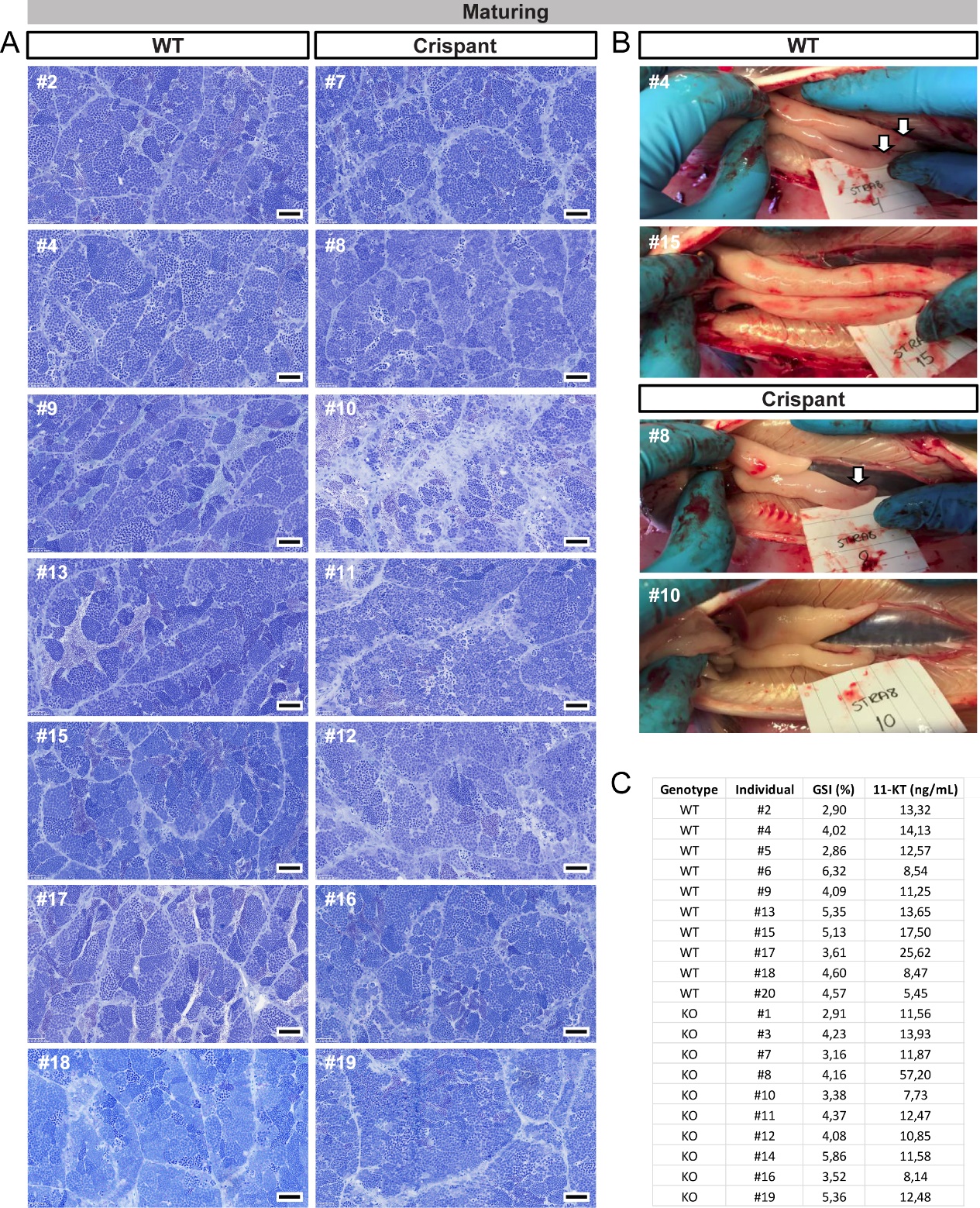


**Supplementary Figure 1. Overview of all maturing males studied at sampling from May 2017.** Microscopical evaluation of maturing WT and *stra8* crispant testis tissue. Scale bar, 50 µm. **(B)** Representative gross morphology of maturing WT and *stra8* crispant testes. White arrows indicate testis tissue areas showing grey coloration, characterized microscopically by the absence/low number of spermatozoa and high number of type B spermatogonia and spermatocytes. **(C)** For each WT and *stra8* crispant fish the sample number, genotype, gonado-somatic index (GSI) and level of plasma 11-ketotestosterone (11-KT) are shown. WT, wild-type; Crispant, *stra8* crispant.


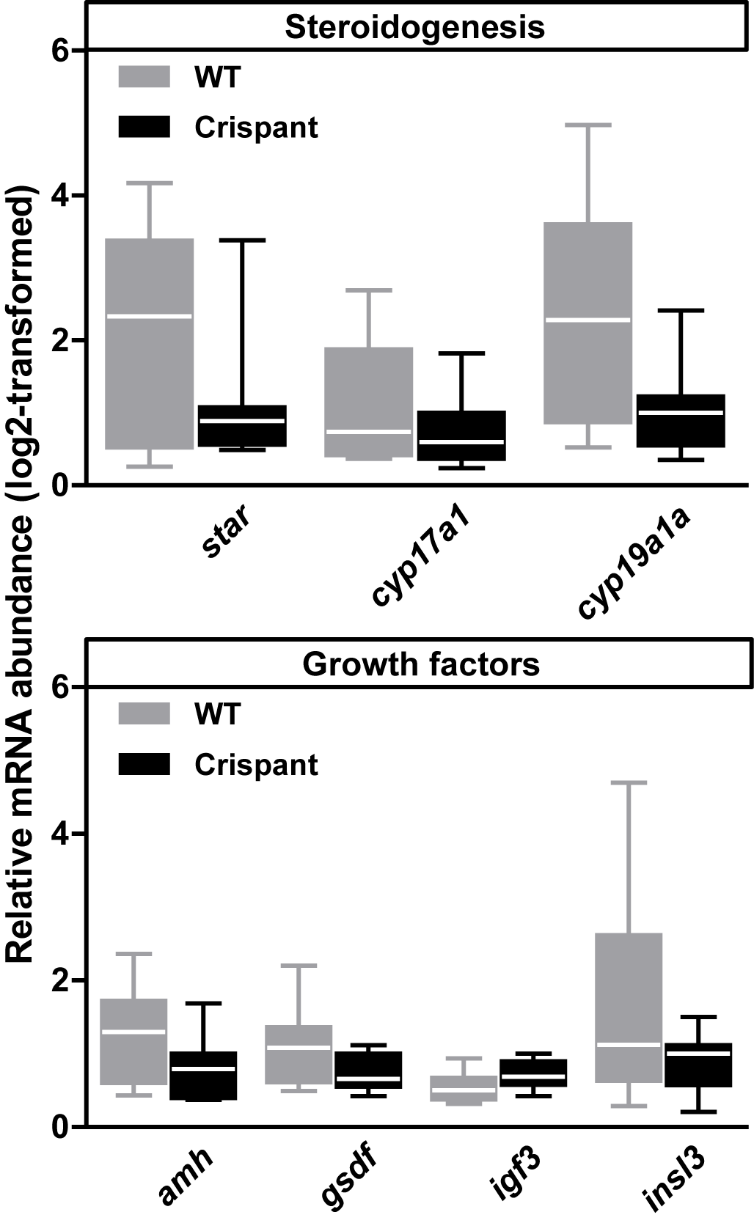


**Supplementary Figure 2. Testicular transcript levels of selected steroidogenesis and growth factors genes in maturing wild-type control and *stra8* crispants.** Transcript levels are shown as mean ± SEM (N = 7-10) and expressed relative to *ef1a* expression. WT, wild-type; Crispant, *stra8* crispant.


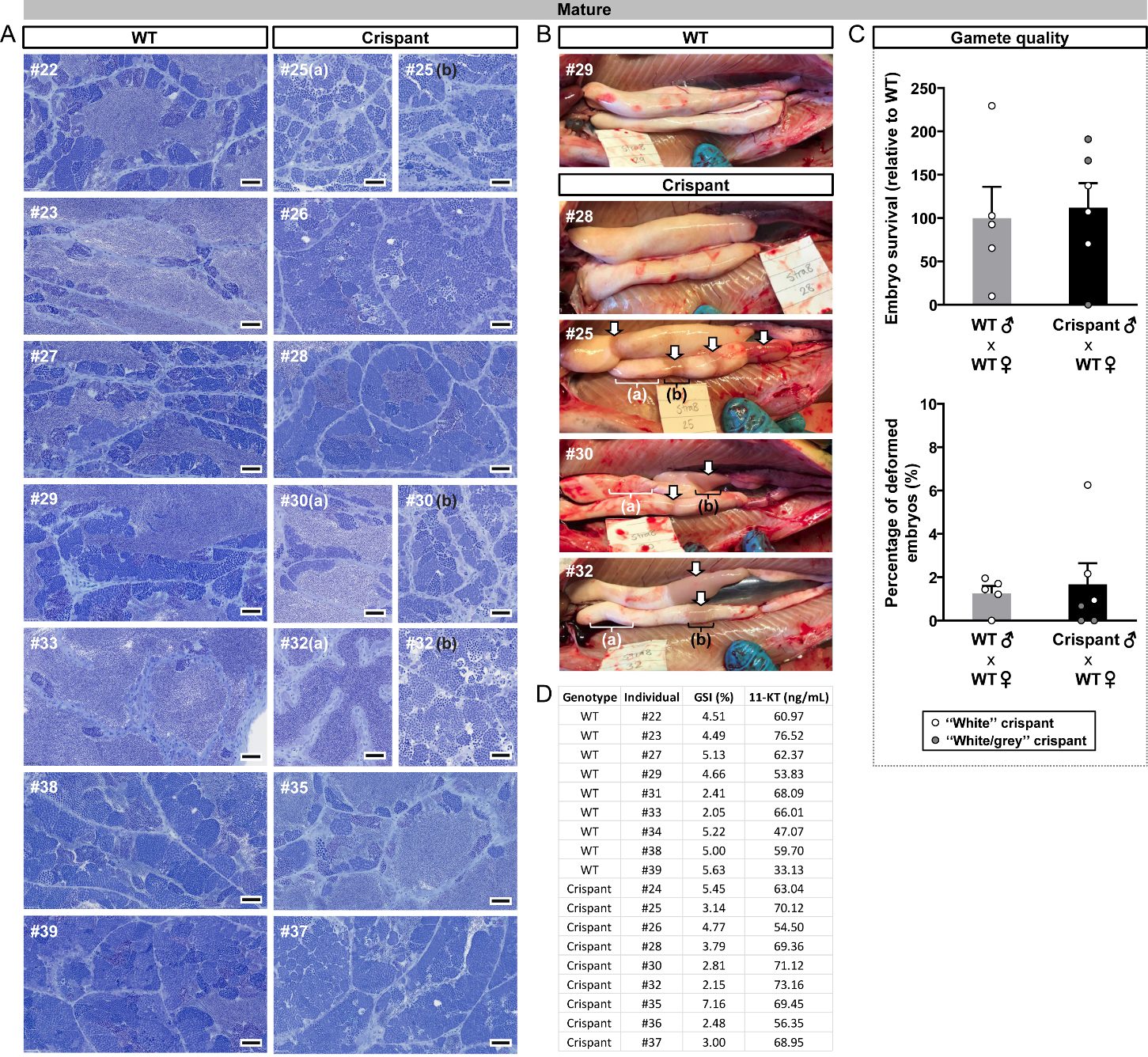


**Supplementary Figure 3. Overview of all mature males studied at sampling from September 2017.** **(A)** Microscopical evaluation of mature WT and *stra8* crispant testis tissue. For individuals #25, #30 and #32 two different testis tissue areas (a and b) are shown due to the different coloration pattern observed, as illustrated in B. Scale bar, 50 µm. **(B)** Representative gross morphology of mature WT and *stra8* crispant testes. White arrows indicate testis tissue areas showing grey coloration, characterized microscopically by the absence/low number of spermatozoa and high number of type B spermatogonia and spermatocytes. **(C)** Embryo survival and development analyses of WT eggs (N = 1) fertilized with WT (N = 5) and *stra8* crispant (N = 6) sperm. Sperm samples were used to individually fertilize ~500-1000 eggs per cross. Data are shown as mean ± SEM and, in the embryo survival panel, expressed relative to the WT control group (which is set at 100). White and grey dots indicate *stra8* crispant individuals showing only white and white/grey testis tissue areas, respectively. **(D)** For each WT and *stra8* crispant fish the sample number, genotype, gonado-somatic index (GSI) and level of plasma 11-ketotestosterone (11-KT) are shown. WT, wild-type; Crispant, *stra8* crispant.


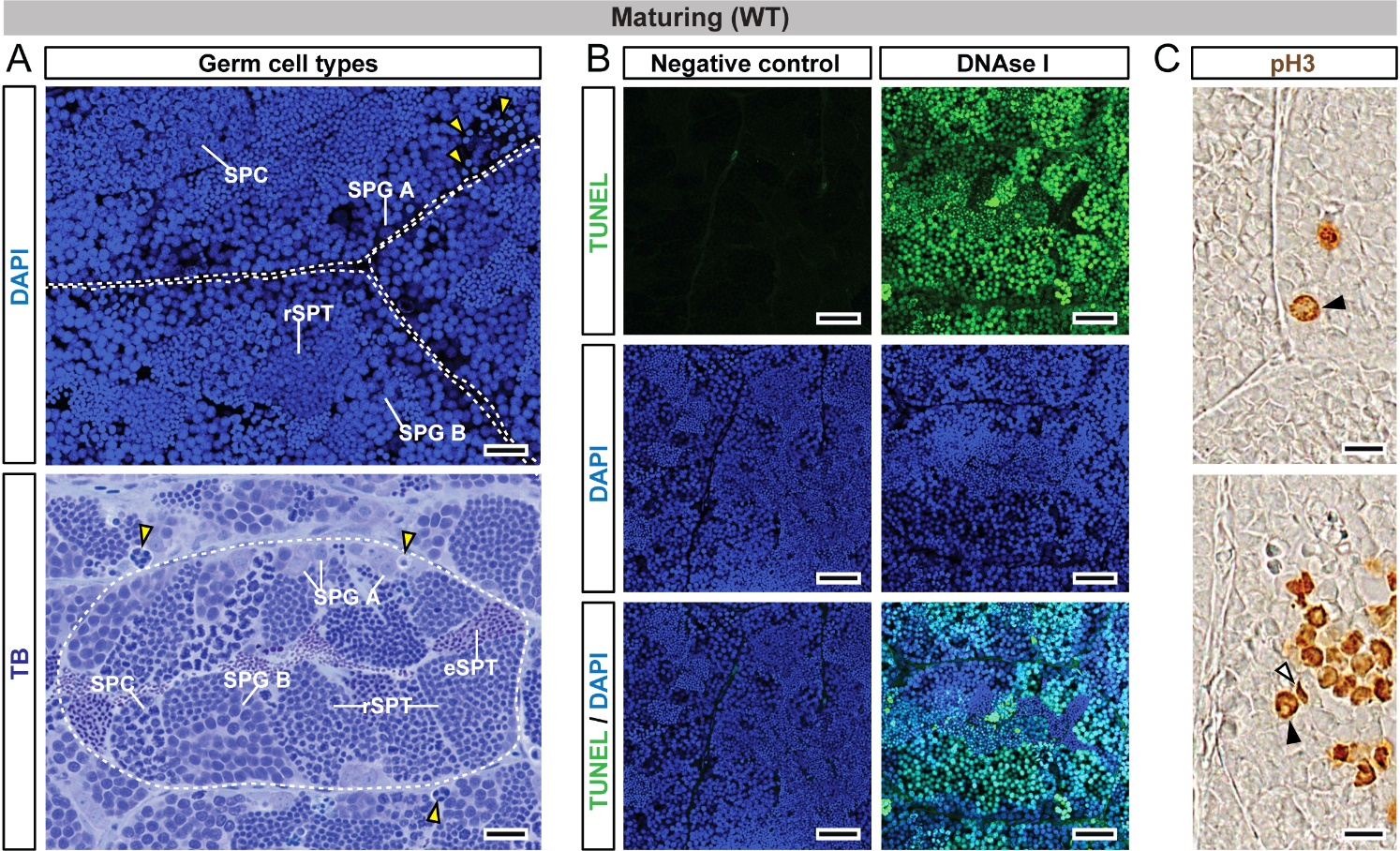


**Supplementary Figure 4. (A)** Identification of different germ cell types in DAPI- and toluidine blue (TB)-stained testis tissue collected form maturing, wild-type (WT) fish. Yellow arrowheads indicate apoptotic germ cells. Scale bar, 20 µm. SPG A, type A spermatogonia; SPG B, type B spermatogonia; SPC, spermatocytes; eSPT, elongated spermatids; rSPT, round spermatids. **(B)** Representative images of tissue subjected to TUNEL analysis. Negative (TUNEL reaction mixture without enzyme solution) and positive (recombinant DNase I) controls were developed according to the manufacturer’s indications. Green staining indicates fragmented DNA in cells undergoing apoptosis (TUNEL+) and blue staining indicates DNA (DAPI counterstain). Scale bar, 30 µm. **(C)** Examples of nuclei of proliferating (pH3+) type A undifferentiated spermatogonia (black arrowheads) and of Sertoli cells (white arrowhead). Scale bar, 15 µm.

## Supplementary Tables

**Supplementary Table 1. Sequences of primers and TaqMan probes used in gene expression analyses by qPCR.**

| **Target gene** | **Forward primer** | **Reverse primer** | **Transcript accession** | **Gene ID** |
| --- | --- | --- | --- | --- |
| *egr1* | ATGTTGCTCCACTCAGCAGG | GTTAGAGCAGCCCTTACCTCC | NM_001141824.1 | 100196802 |
| *dusp4* | CTCAGCCTTGCTCAATGTGTC | TCTTTCACCGAGTCGATAAACTCAA | XM_014129145.1 | 106563492 |
| *lin28a* | CCAGGGAGAGCTCTGATTGAT | TGGATAGATGTGTCTCTCTGTGC | XM_014177858.1 | 106588661 |
| *ret* | CGTGCGGGAAAAACCGATTC | TGGCCCCATAATCGCCAATC | XM_014199328.1 | 106604566 |
| *nanos2* | GGAGAAACTGTGTGACGTTGC | CTCAGGCTCTCAGTCCCGAT | XM_014178458.1 | 106588910 |
| *upp1* | AGGCCTATGAGTCTGGGGTT | ACGGCCGCTTTCAGACC | NM_001141741.1 | 100196719 |
| *pou5f1* | CGCAGACAGAAAGGCAAG | GGTCAGGTGACCCACCAGT | XM_014136460.1 | 106567323 |
| *dmrt1* | AGAATGAACCCACCGGAGAC | AATATGTTTGGACGTGGGTGGA | XM_014172771.1 | 106586001 |
| *sall4* | GGCTAATTTTACAACCCGAAGAA | AGAACGCCATTTTCTGACGA | XM_014136031.1 | 106567117 |
| *ccnd2* | ACCAACACAGAGGTGGACTG | TGCAGGCTACTGGCCAAC | XM_014153345.1 | 106576262 |
| *piwil1* | ACGACTACGCACCCAAACTC | TGTAGTGGGTTGGGGAGACA | XM_014171836.1 | 106585526 |
| *dazl* | GGCATCTGCAAAGGATATGGA | TTCAGTTTCCGCCCTTTAAAAC | XM_014178361.1 | 106588867 |
| *kit* | GTTGCCTGCTGGAGATTTCTTG | AAGGGTCACACATACGCTACAG | XM_014168994.1 | 106584118 |
| *dmc1* | CTTGGGGGAGAGTTTCGGAC | CGGGCGTATAACACATTGTCG | XM_014179660.1 | 106589544 |
| *prdm9* | GGAGGTTAGGACATCAGCCA | GCTTTTGTAGATCACCCAGGA | NM_001173912.1 | 100380788 |
| *ccna1* | AGAGCCTCGCCCTGTATGT | AATCTCTGCCAGGGTGTAGC | XM_014129756.1 | 106563854 |
| *sycp3* | AGTTGGATTGGGGAACGAGG | TGCCTTTGTGCATACTGGGT | XM_014125085.1 | 106561293 |
| *spo11* | TCTAGCTACGTCCAAGGGCT | ACAATGTTCCTGATCCCGCC | XM_014135099.1 | 106566748 |
| *rec8* | TCAGCAGATGGTCTCCTATGG | TCAGGAGGTACGAGGACACC | XM_014181058.1 | 106590253 |
| *sox30* | GTTCATGGTCTGGGCGAGAA | ATCCAACCTGGGAACTCCTG | XM_014136757.1 | 106567484 |
| *vasa* (*ddx4*) | GTTTGCCCATGGAACTTGTGT | GCACATTGCAGCCCTTCAGT | NM_001279129.1 | 101448052 |
| *star* | ATGACCCCAACAAGACCAAG | GGGATCCAGCCCTTTAAATC | XM_014171084.1 | 100136492 |
| *cyp17a1* | TCCCATGGCTACAGGTCTTC | CTGCTTTAGGAGACGCAGGT | XM_014154002.1 | 106576693 |
| *cyp19a1a* | TCAAACAGAACCCTGACGTAG | GCTCCCTTTCACCTATAGCAGTGT | XM_014127472.1 | 106587170 |
| *amh* | CAGTCACTCTCTGCAGCCTTACAA | CAACATTGAATCTCCATTTCAGTTTAC | NM_001123585.1 | 100136452 |
| *gsdf* (*dsl1l*) | GGCAGCATTTCAGACCACTA | GACAAAGCAGTGGCTGTACC | XM_014138924.1 | 106568506 |
| *igf3* | GACCGACCGACAAGATGCA | GCAAGGCACAATATGGAGTACA | XM_014146080.1 | 106572168 |
| *insl3* | CTCCGGAGCTTGGACAACAC | AGTCCTCAGGTTGGCAAATTGAT | MF062497.1 | N/A |
| *ef1a* | CCCCTCCAGGACGTTTACAAA | CAGACGGCCCACAGGTACA | NM_001123629.1 | 100136525 |

**TaqMan probes**

| *cyp19a1a* | FAM-ACAGCTCCTAGAAGAGAT-MGB |
| --- | --- |
| *vasa* (*ddx4*) | FAM-AGCACCGGTCACACCATTCGTGAG-TAMRA |
| *amh* | 6FAM-TTTGCCCTCGGGTTGCTTTCCTGTT-TAMRA |
| *insl3* | 6FAM-AACATAGCACCTTCAGTGTGCATCCTCTGG-TAMRA |
| *ef1a* | FAM-ATCGGTGGTATTGGAAC-MGB |
